# Catalyst State Diagnosis of Three-Way Catalytic Converters Using Different Resonance Parameters—A Microwave Cavity Perturbation Study

**DOI:** 10.3390/s19163559

**Published:** 2019-08-15

**Authors:** Carsten Steiner, Vladimir Malashchuk, David Kubinski, Gunter Hagen, Ralf Moos

**Affiliations:** 1 Bayreuth Engine Research Center (BERC), Department of Functional Materials, University of Bayreuth, 95447 Bayreuth, Germany; 2 Ford Research and Innovation Center, Dearborn, MI 48124, USA; dkubinsk@ford.com

**Keywords:** ceria, oxygen storage capacity (OSC), three-way catalyst (TWC), exhaust gas aftertreatment, microwave cavity perturbation, radio frequency, gasoline engine, resonant frequency, quality factor, on-board diagnosis (OBD)

## Abstract

Recently, radio frequency (RF) technology was introduced as a tool to determine the oxygen storage level of a three-way catalyst (TWC) for gasoline vehicles. Previous studies on the investigation of commercial catalysts mostly use only the resonant frequency to describe the correlation of oxygen storage level and RF signal. For the first time this study presents a comparison under defined laboratory conditions considering both, resonance frequency and also the quality factor as measurands. Furthermore, various advantages over the sole use of the resonant frequency in the technical application are discussed. Experiments with Ø4.66’’ catalysts and Ø1.66’’ catalyst cores with alternating (rich/lean) gas compositions showed that the relative change in signal amplitude due to a change in oxygen storage is about 100 times higher for the inverse quality factor compared to the resonant frequency. In addition, the quality factor reacts more sensitively to the onset of the oxygen-storage ability, and delivers precise information about the necessary temperature, which is not possible when evaluating the resonant frequency due to the low signal amplitude. As investigations on aged catalysts confirm, the quality factor also provides a new approach to determine *operando* the ageing state of a TWC.

## 1. Introduction

In order to meet the stringent emission regulations of modern automobiles, efficient exhaust gas aftertreatment systems are necessary. Three-way catalysts (TWC) prevail for gasoline vehicles as they allow converting unburned hydrocarbons (HC), carbon monoxide (CO), and nitrogen oxides (NO_x_) if the engine is stoichiometrically operated [1,2]. Modern TWCs are composed of active metals (Pt, Rh, and Pd) and promotors (ceria-zirconia) on an alumina support. The nanocrystalline ceria serves as an oxygen storage material to buffer lean-rich variations of the engine [2,3,4]. 

For a high conversion of raw engine-out emissions especially during transient driving conditions, it is useful to determine the actual amount of stored oxygen on the TWC. Former investigations in laboratory setups as well as in engine test benches showed that the oxygen storage level of a TWC can be monitored *operando* by radio frequency (RF) technology, which is often also denoted as microwave technology [5,6,7]. Here, the canning of the catalyst serves as a cavity resonator, in which standing electromagnetic waves occur at discrete frequencies (modes). The resonant frequencies, quality factors, and amplitudes of the observed modes depend on the geometry and on the dielectric properties of the cavity volume. In case of TWCs, ceria-zirconia with high specific surface serves as the oxygen storage component. While oxygen is stored under lean exhaust conditions by oxidation of Ce_2_O_3_ to CeO_2_, it is, in return, released under rich conditions by reducing Ce^4+^ to Ce^3+^ again. This is equivalent to the formation of oxygen vacancies [8,9]. From ceria defect chemistry it is known that the reduction-caused formation of oxygen vacancies goes along with an increased electronic conductivity, as a result of the small polaron hopping mechanism of electrons localized at the Ce^3+^ [8,9,10,11,12,13]. Thus, the dielectric properties of ceria-zirconia change with its oxygen deficiency and the RF-based technology is sensitive to the actual oxygen storage level. 

Former investigations using the resonant frequency as RF signal show that the RF signal can be used to control precisely the engine air-fuel-ratio without lambda probes and for On-Board diagnostics (OBD) purposes [14]. Even the aging state of the TWC can be determined [15], since the resonant frequency amplitude is sensitive both to the oxygen storage capacity (OSC) above the light-off temperature and to the catalyst surface area-dependent water adsorption at low temperatures below 100 °C. Furthermore, results prove that effects of H_2_O and CO_2_ can be neglected above catalyst light-off temperature [16], and studies using different hydrocarbons showed that the point of optimal conversion can be determined by the resonant frequency signal more precisely than by a classical lambda probe [17].

Besides TWC, selective catalytic reduction (SCR) systems are also promising applications for RF-based control and monitoring [18]. Even for diesel particulate filters (DPF) [19,20,21,22], as well as for lean NO_x_ traps (LNT) [23,24] and more recently for gasoline particle filters (GPF) with three-way catalyst coating [25], it could be applied. Here, firstly the quality factor was introduced to determine the oxidation state of the TWC coating during transient engine tests. Radio frequency technology was also applied for oxygen storage level determination of ceria powders, the base material of the TWC oxygen storage component [26,27].

The microwave technology for exhaust gas aftertreatment systems is based on the cavity perturbation theory. With this method, dielectric material properties can be determined in a contactless manner by measuring the scattering parameters of an electromagnetic resonator. The characterization of materials with such systems is easiest possible using single-port-resonators with only one coupling element (port). The coupling elements are denoted as antenna in the following. Here, the reflection coefficient *S*_11_ is measured. It provides information about the power that is reflected back to the source port. Other setups, using two-port-resonators (two antennas) allow a more precise determination of resonant parameters. With this setup, additional scattering parameters, like the transmission coefficient *S*_21_, which describes the power that is transmitted from one port to another [28,29,30], can be measured. 

So far, studies about RF technology for TWC have been focusing on the correlation of oxygen storage and resonant frequency as the sole measurand, obtained from the reflection signal *S*_11_. As a more extended approach, this study deals with the determination of the oxygen storage level by considering also the quality factor and will focus on highlighting differences and advantages of both methods. To characterize the radio frequency signal, laboratory experiments on full-size Ø4.66’’-catalysts at constant operation temperature were conducted, as well as light-off experiments with controlled temperature ramps on smaller Ø1.66”-bore cores. Both, fresh and aged catalyst were investigated with the smaller resonator geometry. As a much higher signal-to-noise-ratio (SNR) can be obtained when working with transmission mode (*S*_21_) [29,30,31], in this work, both RF-parameters were calculated from the S_21_ scattering parameters.

## 2. Materials and Methods

**Figure 1:**
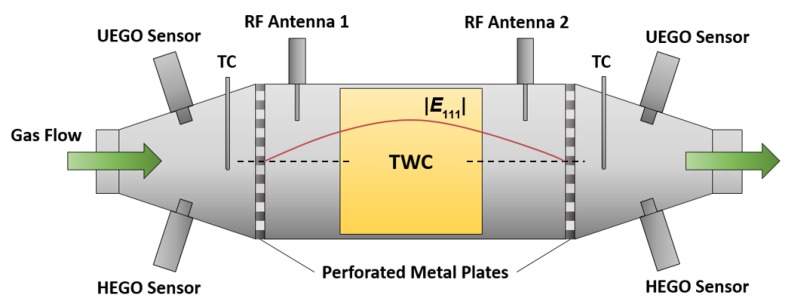
Setup of the cavity resonator for catalyst experiments.

For the catalyst experiments, a commercial TWC was placed in the middle of a catalyst housing. Figure 1 gives a general and schematic overview about the cavity resonator setup for the TWC with the corresponding sensors. The cylindrical resonator comprised a stainless steel canning terminated by perforated metal plates at both the inlet and the outlet side. Two microwave coupling elements (antennas) were applied upstream and downstream of the catalyst brick to couple electromagnetic energy into and out of the cavity resonator. In addition, a binary heated exhaust gas oxygen (HEGO) sensor, a universal exhaust gas oxygen (UEGO) sensor, and a thermocouple (TC) were attached at the cones upstream and downstream. In Figure 1, the absolute value of the electric field |*E*_111_| of the TE_111_-Mode along the central axis is also depicted. In case of a symmetric setup, the electric field maximum appears in the center of the TWC, which is therefore the location with the highest RF sensitivity. To deliver a holistic approach about the radio frequency signal, two different catalyst sizes were used in our study. The setup for the bigger 4.66’’-TWC represents real vehicle geometries and demonstrates the effects of the local electric field strength more clearly. On the other side, the smaller geometry with the 1.66’’-TWC enables a better reflection of the flow conditions, particularly for the gas hourly space velocity (*GHSV*) in real vehicles. Both resonator setups that were used in our study are based on the schematic design in Figure 1 and are described in the following.

### 2.1. Setup A (Ø4.66’’) for Experiments at Constant Temperatures

Experiments with a commercial full-size catalyst (setup A: Ø4.66’’ × 10.7 cm) were conducted at constant temperatures between 280 and 550 °C in steps of approximately 50 °C. Here, for the cavity resonator (Ø12.6 cm × 37.5 cm), two coupling elements with a length of 30 mm were positioned symmetrically, each 75 mm away from the perforated metal plates. The complex scattering parameters were recorded with a sample rate of 1 Hz by a vector network analyzer (VNA) Anritsu ShockLine MS46322A that was connected to the antennas with 50 Ω coaxial cables. With the catalyst placed symmetrically in the cavity center, the TE_111_-Mode appears at about 1.2 GHz for this geometry. The stoichiometry of the exhaust composition was analyzed by BOSCH LSF 4.2 (HEGO) and BOSCH LSF 4.9 (UEGO) sensors mounted at the up- and downstream cones and the temperature was measured with type K thermocouples in the center of the resonator axis. Oxygen and temperature sensor signals were recorded by an IOtech Personal DAQ/3000 module at a sample rate of 5 Hz. 

The outer walls of the cylindrical canning and the cones were actively heated and additionally isolated to achieve typical catalyst temperatures despite low gas flows (pre-heated) and to ensure a more homogeneous temperature profile along the TWC axis and perpendicular to it. With this setup, the temperature gradient along the catalyst axis was measured to be always below 15 °C during all experiments and thus the catalyst temperature could be calculated from the mean temperature of the upstream and downstream thermocouples with sufficient accuracy. 

The catalytic converter was fed with synthetic exhaust (total flow: 20 L/min) from a laboratory gas test bench with BROOKS Instrument mass flow controllers. Considering the brick size, this total flow yielded a *GHSV* of about 1000 h^−1^ for setup A. At constant catalyst temperature, the gases were switched in 20 min intervals with 5000 ppm O_2_, 2000 ppm CO, 10 % H_2_O, 10 % CO_2_, 1000 ppm NO, 250 ppm C_3_H_8_ for lean (λ = 1.02), and 2000 ppm O_2_, 6600 ppm CO, 2200 ppm H_2_, 10 % H_2_O, 10 % CO_2_, 1000 ppm NO, 250 ppm C_3_H_8_ for rich (λ = 0.98) feed gas.

### 2.2. Setup B (Ø1.66’’) for Light-off Experiments

We also investigated the behavior of the RF signal during catalyst light-off as the temperature was ramped, including results for both new and thermally aged TWCs. For these experiments, a smaller resonator- and catalyst geometry was used to achieve space velocities that are typical for TWCs during engine operation, and to allow a better mapping of catalyst properties during dynamic measuring conditions. In these tests, the resonator setup comprised a canning (Ø1.75’’ × 18 cm) with 20 mm antennas with a catalyst type TWC (Ø1.66’’ × 7.7 cm) inside. Perforated metal screens were placed at both ends of the cylindrical cavity. The antennas were 14.3 cm apart, and the upstream face of the catalyst located 5.8 cm from the front reflecting screen. The transmission signal of the cavity was reported by an Agilent 1260C VNA. The TE111-Mode appeared at around 3.1 GHz. With a total gas flow of 60 L/min, a *GHSV* of approximately 32000 h^−1^ was achieved. The resonator setup was thermally isolated, but not actively heated at the outer walls. Here, the total thermal energy came from the pre-heated gas flow. For the light-off experiments, the TWC was heated up from room temperature to 600 °C within a controlled temperature ramp (3 K/min). During the heating process, the gas atmosphere (a mixture of N_2_, O_2_, C_2_H_4_, H_2_O, and CO_2_) was alternately switched from lean (λ = 1.01) to rich (λ = 0.99) every 180 seconds. The interval time was chosen to ensure that above the oxygen storage light-off temperature the catalyst was fully oxidized at the end of each lean interval, and in a steady-state reduced condition at the end of each rich interval. The outlined experiments were performed with a fresh TWC as well as with one that was hydrothermally aged in λ = 1 combustion exhaust gas for 150 h at 890 °C. The gas temperature was taken using thermocouples placed just before the cavity’s front reflecting screen and after the downstream screen. The reported temperature was the mean of these two measurements. Automotive oxygen sensors (both the linear (UEGO) and switching (HEGO) types) were placed upstream and downstream the resonant cavity to monitor the input and output net oxygen concentrations. Additionally, the downstream gas composition was monitored using a MIDAC FTIR and an H_2_ gas analyzer.

### 2.3. Calculation of Resonance Parameters from Spectral Data

**Figure 2:**
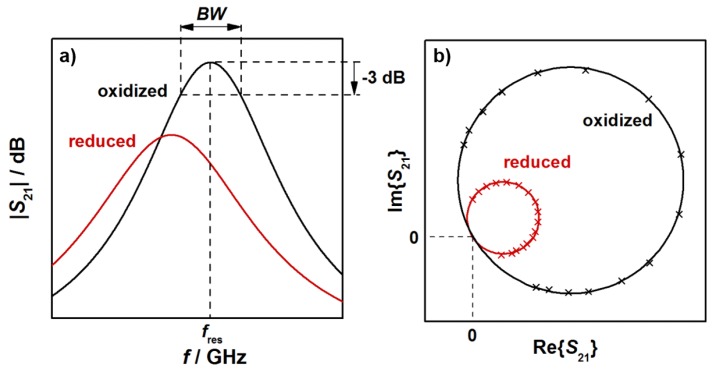
(**a**) Transmission parameter *S*_21_ and (**b**) complex spectrum of the TE_111_ mode for an oxidized and a reduced three-way catalyst.

In this study, resonant frequencies and unloaded quality factors were determined for the TE_111_-mode from the transmission signal *S*_21_, as explained below. Typical radio frequency signals, observed for an oxidized (black) and a reduced (red) three-way catalysts, are shown in Figure 2. The relation between the logarithmic *S*_21_ (in dB) and the frequency *f* of the electromagnetic wave is shown schematically in Figure 2a for a small frequency range around the resonant frequency. Indicated are also the resonant frequency, which is the frequency at the maximum *S*_21_ amplitude, and the bandwidth *BW,* over which the power of the electromagnetic TE_111_ resonance is greater than half of the maximum resonance power at *f*_res_. With both parameters, the loaded quality factor *Q*_l_ can be determined (Q_l_ = *f*_res_/*BW*). As Figure 2a shows, the reduction of the TWC leads to a lower resonant frequency and a broader resonant peak, i.e., to an increase of the bandwidth *BW*. As the relative signal amplitudes of the bandwidth *BW* are typically much greater than for the resonant frequency *f*_res_, changes in *Q*_l_ are dominated by the bandwidth. As a result, the reduction of the TWC leads to lower quality factors. 

It is well known from cavity perturbation theory that the resonant frequency is related to the polarization of the material inside the cavity resonator, while the quality factor or inverse quality factor (1/*Q*_l_) correlates with the corresponding dielectric losses. Thus, the measured changes in these radio frequency signals upon a change of oxidation state of the TWC are the result of corresponding changes in both its material polarization and dielectric losses. These results are also confirmed by measurements with ceria and ceria-based catalyst powders, and in a more defined resonator geometry [26,27] and are well in line with the understanding about ceria defect chemistry [8,9,10,11,12,13].

Figure 2b shows the corresponding complex representation of *S*_21_ with the resonant circles of the oxidized and the reduced catalyst. In the reduced state, the diameter of the resonant circles is smaller. The resonance parameters in this study were calculated based on a fitting method of the resonant circles in the complex plane by a least square regression (as shown in Figure 2b), followed by the transformation of the circle position into the so-called canonical position [31,32]. By an evaluation of the phase (*α*)-frequency (*f*) correlation, the resonance frequency can be obtained at *α* = 0, while the bandwidth is calculated from *BW* = |*f*(*α* = +π/4) − *f*(*α* = −π/4)| [32]. As all points near the resonance are used for the calculation, a precise determination of the resonance parameters is possible with this method [30,31,32,33,34]. With the calculation of the unloaded quality factor *Q*_0_, we additionally eliminated the cavity coupling effects. Since the resonator geometries used in this study are symmetrical, the inverse unloaded quality factor, which correlates to the dielectric losses of the resonator, can be calculated by *Q*_0_^−1^ = *Q*_l_^−1^(1 − 10*^S^*^21,res/20^), with *S*_21,res_ being the amplitude of the transmission parameter in dB at the resonant frequency *f*_res_ [28,29,33]. We have to note here, that only setup A was symmetric. According to the given equations, resonator coupling can be considered and the unloaded quality factor has been determined for the experiments with constant temperatures. For the light-off experiments, only the loaded quality factor was calculated. Nevertheless, in both cases the correlation between TWC material properties and RF parameters can be deduced, since only changes of the resonance parameters are of interest in this study.

## 3. Results and Discussion

### 3.1. Experiments with Setup A (Ø4.66’’) at Constant Catalyst Temperatures

**Figure 3:**
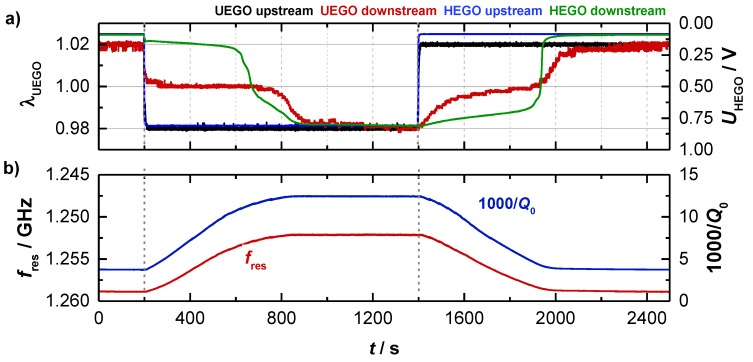
(**a**) Catalyst experiment of the three-way catalyst (TWC) at 400 °C with (**a**) the wideband and binary sensor signals and (**b**) the corresponding radio frequency signals *f*_res_ and *Q*_0_^−1^ of the TE_111_-Mode.

As mentioned above, experiments with a Ø4.66’’-TWC (setup A) were performed to analyze the basic behavior of the resonance parameters. Therefore, the gas conditions were switched alternately from lean to rich at constant catalyst temperature. The basic experiment is shown in Figure 3 for data taken at 396 °C. Shown are (a) the lambda sensor signals and (b) the corresponding RF parameters as derived from the transmission parameter. The signals of the binary (HEGO) and wideband lambda sensors (UEGO) upstream of the TWC reflect the change of the feed gas composition. When the feed gas is switched, the downstream sensors initially indicate a stoichiometric gas composition, as the stored oxygen on the TWC reacts with the rich gases during the lean-rich change or as the reduced TWC stores oxygen during the rich-lean change. When the oxygen storage capacity of the TWC is almost exhausted, it comes to the breakthrough of the feed gas conditions and the sensor signals of the upstream and downstream sensors converge. 

As can be seen in Figure 3b, the resonant frequency *f*_res_ (red) and the inverse (unloaded) quality factor *Q*_0_^−1^ (blue) start both at constant levels (*f*_res_ = 1.259 GHz and *Q*_0_^−1^ = 3.5) at the beginning of the test (0 ≤ *t* ≤ 200 s) when the TWC is fully oxidized. After the feed gas is switched to rich (*t* = 200 s), the resonant frequency *f*_res_ initially decreases continuously. Correspondingly, the opposite reaction (increase) is measured for the inverse (unloaded) quality factor *Q*_0_^−1^. Both curves flatten out when the catalyst becomes strongly reduced, and reach a constant level (*f*_res_ = 1.252 GHz and *Q*_0_^−1^ = 12.4) when the upstream and downstream sensors show identical signals, i.e., when the oxygen storage material is fully reduced at the applied conditions. As mentioned above, the decreasing *f*_res_ indicates a stronger polarization for a reduced TWC, as well as the increasing *Q*_0_^−1^ reports higher losses for this case. These findings are in agreement with the defect chemistry of ceria, which suggests the formation of additional conduction electrons with proceeding reduction of Ce^4+^ to Ce^3+^ [8,9,10,11,12,13]. When switching to lean conditions (*t* = 1400 s), again a continuous change is measured for the radio frequency parameters, until their signals flatten out, when the breakthrough is detected downstream. When the TWC is fully oxidized again, *f*_res_ and *Q*_0_^−1^ reach their initial levels.

The experiment shows that the resonant frequency is sensitive to the oxygen storage level of the TWC and so confirms the findings from previous investigations [14,15,16,17]. Additionally, the results also indicate that the inverse (unloaded) quality factor can be used for state diagnosis of the oxygen storage, because it depends on the increase of conductivity of a reduced TWC. 

In order to obtain a better understanding of the correlation between the oxygen storage level and the microwave parameters, the experiment shown in Figure 3 was conducted at different catalyst temperatures between 280 and 550 °C. This range was chosen since it bounds the light-off temperature for the oxygen storage on the TWC. Moreover, using Equation (1), the oxygen storage capacity (*OSC* in g/L) was determined, which is the amount of stored oxygen in case of a rich-lean switch related to the catalyst volume. Also, the volume-based amount of released oxygen (in g/L) during a lean-rich switch—the so-called and oxygen release capacity (*RSC*)—were determined for all experiments. To determine these parameters, the signal difference between the upstream and downstream wideband lambda probes was integrated [34,35]: Such calculations are used also in other studies, e.g., in [15,36].

(1) $$\mathrm{OSC}\;=\;\frac{p_{0}M_{O_{2}}}{{RT}_{0}}\frac{1\;+\;\frac{x}{4}}{1\;+\;\frac{x}{2}\;+\;\frac{1\;+\;\frac{x}{4}}{y_{O_{2}}}(1-y_{O_{2}})}\mathrm{GHSV}\int_{t_{1}}^{t_{2}}|\lambda_{\mathrm{up}}-\lambda_{\mathrm{down}}|\mathrm{dt}$$

with the pressure *p*_0_ = 1.013 bar and the temperature *T*_0_ = 273.15 K at standard conditions, the molar weight of an oxygen molecule *M*_*O*_2__ = 32 u = 32⋅1.661⋅10^−27^ kg, the universal gas constant R = 8.314 J/(mol K), the signal of the UEGO sensors upstream (*λ*_up_) and downstream (*λ*_down_), as well as the molar H/C-ratio *x* of the fuel and the oxygen fraction $y_{O_{2}}$ of the air, used for the combustion. For laboratory experiments, the theoretical values of *x* and $y_{O_{2}}$ can be calculated from the composition of the synthetic exhaust gas [15]. The integration of the *OSC* was started (*t*_1_) as soon as the upstream UEGO sensor indicates a lean exhaust (*λ*_up_ > 1) and was stopped (*t*_2_), when a defined breakthrough voltage at the downstream HEGO sensor was measured (*U*_down_ < 200 mV). Since the absolute value |*λ*_up_ − *λ*_down_| is used in Equation (1), the *RSC* can also be calculated by using the same equation. Correspondingly, *RSC* integration is started (*t*_1_), when *λ*_up_ < 1, and stopped (*t*_2_) again when *U*_down_ > 750 mV. The reduction and re-oxidation of the catalyst was repeated five times at each constant temperature value studied. As the first cycle was not always reproducible in the experiments, only the last three cycles are part of the data used in this publication.

**Figure 4:**
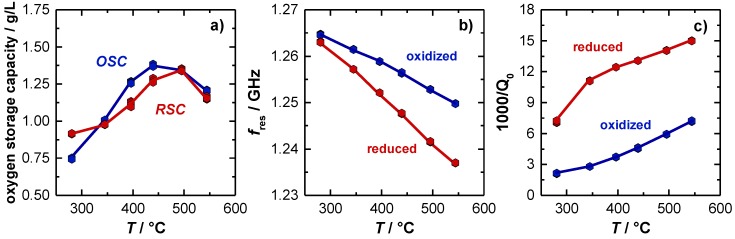
(**a**) Effect of catalyst temperature on oxygen storage and release capacity (*OSC*, *RSC*). Effect of catalyst temperature and oxygen storage level on (**b**) resonant frequency and (**c**) unloaded quality factor. The change in 1000/*Q*_0_ is referred to as amplitude of the (unloaded) quality factor in the following.

Figure 4a shows the temperature dependent *OSC* and *RSC* of the TWC. Here, the *OSC* activity is already observed to start at 280 °C (approx. 0.8 g/L). First, the amount of released and re-stored oxygen increases with higher temperatures and finally stagnates with 1.35 g/L at a temperature of about 500 °C. The corresponding resonant frequencies *f_res_* and the inverse (unloaded) quality factors *Q*_0_^−1^ for the oxidized and the (fully) reduced TWC are shown in Figure 4b,c. For the resonant frequency, the typical almost linear temperature dependency with negative slope was observed, which can be explained firstly by the thermal expansion of the cavity and secondly by a stronger polarization of the catalyst material with higher temperatures. Considering the oxidation state of the catalyst, also an increase of the signal amplitude with higher temperatures is observed. Both, the measured *OSC*/*RSC* and the resonant frequency signal is well in line with previous literature [14]. Focusing now on the unloaded quality factor, 1/*Q*_0_ has a clear upward tendency, which can be explained by increasing losses inside the cavity resonator at higher temperatures. Additionally, the measured losses are significantly higher when the catalyst is reduced. This correlates also well with the defect chemistry of ceria, or ceria-zirconia respectively, as more oxygen vacancies can be formed at higher temperatures. This eventually leads to more free electrons and hence to higher losses inside the resonator [9,12,37,38].

In the experiment, significant differences between the signal of the resonant frequency and the inverse (unloaded) quality factor are observed. At low catalyst temperatures (<300 °C), only a very small amplitude for an oxidized and reduced catalyst is measured for the resonant frequency (2 MHz), while the reciprocal quality factor amplitude is already very large at this temperature, and increases even more to its maximum at 350 °C. Additionally, for higher temperatures the amplitude of the inverse quality factor remains constant (Δ(1000/*Q*_0_) = 8.2) and seems to be independent of temperature. This is completely different to the resonant frequency *f*_res_, as explained above. The experiment suggests that the (unloaded) quality factor might be best suited to detect the oxygen storage level, especially in the low temperature range, when changes in the oxygen storage between the lean and rich gas states begin to occur. 

**Figure 5:**
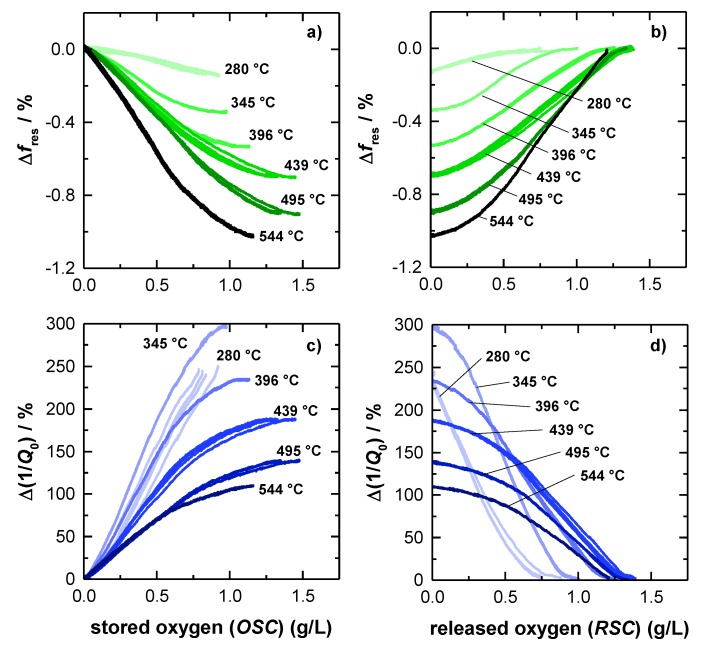
Correlation of the relative resonant frequency amplitude and the oxygen storage level during (**a**) rich-lean switch and (**b**) lean-rich switch. Correlation of the relative amplitude of the unloaded quality factor and the oxygen storage level during (**c**) rich-lean switch and (**d**) lean-rich switch.

To investigate this assumption more in detail, Figure 5 shows the correlation of the oxygen storage level and the radio frequency-derived signals for each investigated catalyst temperature. Therefore, the relative changes of resonant frequency Δ*f*_res_ and (unloaded) quality factor Δ(1/*Q*_0_) referred to a fully oxidized TWC were determined, as this represents a well-defined catalyst state. Accordingly the relative changes of the resonant frequency and the inverse (unloaded) quality factor can be obtained by Δ*f*_res_ = (*f*_res,meas_ − *f*_res,ox_)/(*f*_res,ox_) and Δ(1/*Q*_0_) = (1/*Q*_0,meas_ − 1/*Q*_0,ox_)/(1/*Q*_0,ox_), with the measured resonant frequency and quality factor *f*_res,meas_ and *Q*_0,meas_, and the referred RF parameters *f*_res,ox_ and *Q*_0,ox_ of the fully oxidized TWC at the corresponding temperature.

The relative frequency change Δ*f*_res_ as a function of the amount of oxygen, released during lean-rich-switch, is given in Figure 5a. Similarly, Figure 5b shows the relation between the relative f_res_^-^ signal and the amount of stored oxygen, when the atmosphere was changed the other way around from rich to lean. The corresponding relative changes of the inverse (unloaded) quality *Q*_0_^−1^ factor are presented in Figure 5c,d. 

The amount of stored and released oxygen was calculated by the integration method as explained above (Equation (1)). As a result, the amount of released oxygen (Figure 5a,c) is based on the *RSC* integration, while the amount of stored oxygen is calculated from *OSC* balance (Figure 5b,d). For both resonance parameters, the relative signal amplitudes are referenced to a (fully) oxidized TWC, as this represents a well-defined oxygen storage with no oxygen vacancies. The reduction degree under rich conditions, instead, highly depends on the gas composition and on the catalyst temperature. 

In Figure 5a the resonant frequency starts at 0 %, which is, as explained, equal to a fully oxidized catalyst. As the reduction of the oxygen storage proceeds, *f*_res_ decreases, ending in the maximum amplitude at each temperature, when the total *RSC* is used. The opposite behavior can be observed for rich-lean switches (Figure 5b). Here, the resonant frequency increases, while the initially reduced TWC is gradually oxidized, and ends again at 0 %, when the catalyst is fully re-oxidized. Also, in this case, the results for the resonant frequency is well in line with previous literature and matches our experiences [15,16,17].

The experimental results also show that the maximum relative amplitude of *f*_res_ is increasing continuously with higher temperatures and reaches its maximum of ~1% at the highest catalyst temperature of 544 °C. Additionally, the resonant frequency appears to be mostly linear, for only weakly reduced catalysts and becomes progressively nonlinear in case of a strongly reduced catalysts at high temperatures (>400 °C). Looking at the resonant frequency more closely, a slightly s-shaped dependency of the oxygen storage level is observed, with the highest sensitivity at 50% of total capacity (e.g., 345 °C and 396 °C). This s-shaped behavior can be explained by the electrical field distribution of the TE_111_-Mode, which has its maximum in the center of the symmetric geometry (see also Figure 1). Because storing/releasing oxygen proceeds from the catalyst front to the backside, the maximum RF-sensitivity must be expected when the oxygen storage process occurs in the middle of the catalyst.

As explained previously, Figure 5c,d show the relative change of the inverse (unloaded) quality factor depending on the TWC oxygen storage level. In Figure 5c, starting with a fully oxidized TWC again, the relative change of *Q*_0_^−1^ is found to be increasing with a proceeding release of oxygen, which can be attributed to increasing losses, as explained above. In contrast to the resonant frequency, the relative change of *Q*_0_^−1^, is decreasing with higher temperatures. As the absolute signal amplitude of the inverse unloaded quality factor remains unaffected by catalyst temperature (Figure 4c), this effect is due to the general increase of dielectric losses of the (fully) oxidized catalyst (see also Figure 4c), which is the referenced catalyst state of Figure 5. Even at 544 °C, the relative change, observed for *Q*_0_^−1^ is higher by a factor of at least 100 compared to the *f*_res_-signal and is even higher at lower catalyst temperatures. Regarding this point, the quality factor has a clear advantage over the resonant frequency.

Comparing the graphs of resonant frequencies and inverse quality factors in Figure 5, the experiments also confirm that both resonance parameters look similar. Also, in case of the quality factor, a non-linear loss in sensitivity is observed for a highly reduced TWC at higher temperatures, as well as the slight s-shape that originates from the local electric field distribution. In this context, the quality factor can deliver at least the same information about the oxidation state of the catalyst. Even more: For the relative *Q*_0_^−1^-signal, the highest absolute (Figure 4c) and relative amplitude (Figure 5c,d) were measured at 345 °C. Below this temperature (280 °C), the absolute and relative amplitude is smaller, but as Figure 5c,d shows quite nicely, the gradient is very steep at both temperatures. Thus, the correlation of the oxygen storage level and the inverse quality factor shows clearly that at low catalyst temperatures even small changes in the oxidation state of the TWC have a high impact on the inverse quality factor. This confirms that the quality factor is indeed superior to the resonant frequency in this this temperature area, because of its by far higher sensitivity. To validate the data observed for the big 4.66’’ geometries and to further investigate the correlation of catalyst light-off and inverse quality factor, additional measurements were conducted also for smaller Ø1.66’’ TWC bricks in this study.

### 3.2. Light-off Experiments with Setup B (Ø1.66’’)

In the light-off experiments, the smaller Ø1.66’’-catalysts (setup B) were heated up with a controlled temperature ramp from room temperature to 600 °C. During the heating process, the gas composition was changed alternatingly from lean to rich. Similar measurements were also conducted in [15]. The experiments were performed for a fresh and an aged TWC. 

**Figure 6:**
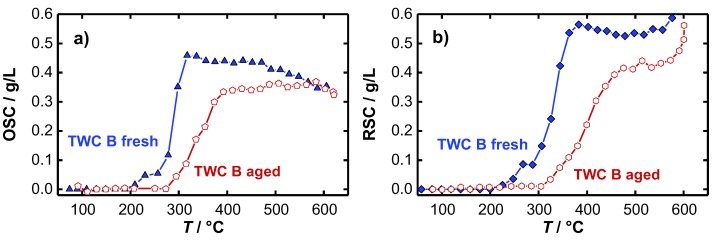
Temperature-dependent (**a**) *OSC* and (**b**) *RSC* of the fresh and aged TWC B.

The temperature dependent *OSC* and *RSC* of the fresh and the aged TWC were calculated according to Equation (1) and are presented in Figure 6. As expected, for both the fresh (a) and the aged (b) catalyst it is found that no oxygen is stored at low temperatures. A first oxygen storing/releasing effect appears at approximately 250 °C for the fresh TWC, and not until 300 °C for the hydrothermally aged catalyst. In addition to an increase of the temperature, necessary for the activation of the oxygen storage, also a decrease in the oxygen storage capacity for the aged TWC was observed. Both findings represent typical aging effects, which are addressed in literature to various mechanisms occurring during the hydrothermal aging process. According to [39,40,41], the active surfaces of the oxygen storage material decrease as a result of sintering processes that highly reduces the amount of useable oxygen for oxidation reactions. Also, the loss of noble-metal dispersion and migration or encapsulation of noble metal strongly deteriorate the catalyst performance [42,43,44,45], and shifts the catalyst light-off to higher temperatures. We note that for each catalyst the calculated *RSC* value is greater than the calculated *OSC*. This presumed artifact is not fully understood. The water-gas shift reaction may influence the balancing method. A qualitative statement about catalyst aging and oxygen storage activity can be made in any case. 

**Figure 7:**
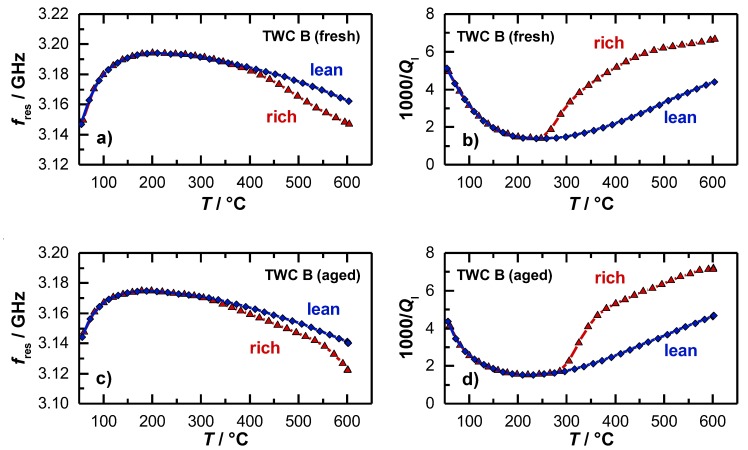
Radio frequency-derived signals as a function of the catalyst temperature during light-off experiments: (**a**) Resonant frequency and (**b**) inverse loaded quality factor for an oxidized and reduced TWC (fresh); (**c**) resonant frequency and (**d**) inverse loaded quality factor for an oxidized and reduced TWC (aged).

For both investigated catalysts, also the temperature dependent resonance parameters were reported. The results for the resonant frequency and the loaded quality factor are shown in Figure 7a,b for the fresh catalyst and in Figure 7c,d for the aged catalyst. For the resonant frequency (Figure 7a), the typical behavior was found that had also been observed in [15] and [36]. When the heating ramp is started, the resonant frequency increases strongly first. This is due to water that desorbs from the catalyst surface, which has a dominating impact on the polarization. When the maximum resonant frequency is reached at approximately 230 °C, *f*_res_ decreases with higher temperatures. This has also been found in the experiments of the 4.66’’-TWC before. This effect can be explained again by the thermal expansion of the resonator geometry and by the increasing material polarization. Above 300 °C, the resonant frequencies for the catalyst under lean (blue) and rich gas (red) diverge, which indicates an active oxygen storage. However, the derivation of a clear temperature, from which the oxygen storage is active, is not directly possible with the resonant frequency, as the amplitude is very small in this temperature range, which correlates again well with the findings of the former experiment (Figure 5). 

In contrast to the resonant frequency, the inverse (loaded) quality factor in Figure 6b decreases at the beginning of the heating ramp, which is also in this case related to the water desorption, as the presence of water on the catalyst surface strongly affects the dielectric losses and attenuation inside the cavity resonator. For the inverse quality factor, a minimum also occurs at 230 °C, with an increasing trend at higher temperatures, which can again be explained by a thermal activation of dielectric losses of the catalyst material. 

The most interesting point in the experiment is the signal of the inverse loaded quality factor 1/*Q*_l_, when the oxygen storage level of the TWC begins to change. A major difference can be observed between the two resonance parameters. While the resonant frequencies of the oxidized and reduced TWC diverge only slightly, the inverse quality factors differ greatly as soon as the oxygen storage becomes activated. The quality factor signal indicates that a first reduction and re-oxidation of the ceria is possible between 250 and 265 °C (fresh TWC), which is well in line with the findings of the *OSC*/*RSC* measurements (Figure 6). At this temperature, the resonant frequency does not respond to oxidation state of the TWC at all. Again, these results demonstrate that only the quality factor delivers information about the onset of the change in the oxidation storage level as the temperature is increased.

The light-off experiments were also conducted for aged catalysts. The results for the resonant frequency and inverse loaded quality factor are presented in Figure 7c,d. In general, both parameters show the same basic profile, but some differences can be found related to the aging state of the catalyst. Firstly, the smaller increase of the resonant frequency and decrease of the inverse quality factor at the beginning of the heating ramp indicate that less water is absorbed. This can be explained by the loss of surface area during the aging process. Secondly, the smaller amplitude of the change in resonance frequency between the rich and lean gases also suggests that the total oxygen storage capacity was reduced by catalyst aging, as measured in [15]. And as the third point, the inverse (loaded) quality factor shows that a first reduction of the oxygen storage material can be seen only above 300 °C for the aged catalyst, which is about 50 °C higher compared to the fresh catalyst, which accurately reflects the results for the *OSC*/*RSC* of both catalysts (Figure 6). 

As a result, the experiments demonstrate that the actual oxygen storage level of a TWC cannot only be determined by the inverse quality factor, but the quality factor is the more suitable parameter for operation near the catalyst light-off temperature. In contrast to the resonant frequency, with the inverse quality factor the activation of the oxygen storage can be detected accurately, and even the aging state can be determined more precisely. Together, both parameters, resonant frequency and quality factor, can deliver a more complete information about catalyst properties, especially related to the current aging state of the catalyst. From this point of view, a parameter set from both signals opens new possibilities for the catalyst state diagnosis.

## 4. Conclusions

In this study, a deeper analysis of radio frequency technology for state diagnosis of three-way catalytic converters was presented. In our new approach, two resonance parameters, resonant frequency, and (inverse) quality factor were determined, differences in both signal were highlighted, and advantages of both methods are discussed detailed.

As well as the resonant frequency, also the inverse quality factor delivers information about the oxygen storage level of a TWC. The amplitude of the resonant frequency was observed to be very small at catalyst temperatures around catalyst light-off. Here, the quality factor clearly shows advantages over resonant frequency. It responds much more sensitive to the activation of the oxygen storage component, because the losses in the resonator strongly increase, as soon as the reduction of ceria occurs. With the quality factor, also the temperature that is necessary for the onset of the oxygen storage ability, can be easily determined. Moreover, the increase of this characteristic temperature due to catalyst ageing can be observed and thus the quality factor offers an additional possibility to evaluate the ageing state of the catalytic converter in the vehicle.

Finally, a parameter set, including both resonance parameters, delivers a more precise and detailed picture about processes inside the oxygen storage material of a three-way catalytic converter and opens up new possibilities for the technical application. Also, new approaches to determine the catalyst aging state are available.
